# Associations between Witnessing and Perpetrating Online Hate in Eight Countries: The Buffering Effects of Problem-Focused Coping

**DOI:** 10.3390/ijerph16203992

**Published:** 2019-10-18

**Authors:** Sebastian Wachs, Michelle F. Wright, Ruthaychonnee Sittichai, Ritu Singh, Ramakrishna Biswal, Eun-mee Kim, Soeun Yang, Manuel Gámez-Guadix, Carmen Almendros, Katerina Flora, Vassiliki Daskalou, Evdoxia Maziridou

**Affiliations:** 1 Department of Educational Studies, University of Potsdam, 14476 Potsdam, Germany; 2 Department of Psychology, Pennsylvania State University, University Park, PA 16802, USA; mfw5215@psu.edu; 3 Faculty of Social Studies, Masaryk University, 60200 Brno, Czech Republic; 4 Kids and Youth Development Research Unit, Research Center for Educational Innovations and Teaching and Learning Excellence, Faculty of Humanities and Social Sciences, Prince of Songkla University, Muang, Pattani 94000, Thailand; ruthaychonnee.s@psu.ac.th; 5 Department of Human Development and Family Studies, G.B. Pant University of Agriculture and Technology, Pantnagar 263145, Uttarakhand, India; ritu.singh07@gmail.com; 6 Department of Humanities and Social Sciences, National Institute of Technology, Rourkela 769008, India; rkbpsych@gmail.com; 7 Department of Communication, Seoul National University, Seoul 08826, Koreasoeun022@snu.ac.kr (S.Y.); 8 Department of Biological and Health Psychology, Autonomous University of Madrid, 28049 Madrid, Spaincarmen.almendros@uam.es (C.A.); 9 Department of Psychology, Neapolis University Pafos, 8042 Pafos, Cyprus; K.flora@nup.ac.cy; 10 Department of Psychology, Aristotle University of Thessaloniki, 54124 Thessaloniki, Greece; vassilikida@yahoo.gr (V.D.); evamazirid@gmail.com (E.M.)

**Keywords:** online hate, hate speech, bystander, perpetrator, coping strategies, cyber aggression

## Abstract

Online hate is a topic that has received considerable interest lately, as online hate represents a risk to self-determination and peaceful coexistence in societies around the globe. However, not much is known about the explanations for adolescents posting or forwarding hateful online material or how adolescents cope with this newly emerging online risk. Thus, we sought to better understand the relationship between a bystander to and perpetrator of online hate, and the moderating effects of problem-focused coping strategies (e.g., assertive, technical coping) within this relationship. Self-report questionnaires on witnessing and committing online hate and assertive and technical coping were completed by 6829 adolescents between 12 and 18 years of age from eight countries. The results showed that increases in witnessing online hate were positively related to being a perpetrator of online hate. Assertive and technical coping strategies were negatively related with perpetrating online hate. Bystanders of online hate reported fewer instances of perpetrating online hate when they reported higher levels of assertive and technical coping strategies, and more frequent instances of perpetrating online hate when they reported lower levels of assertive and technical coping strategies. In conclusion, our findings suggest that, if effective, prevention and intervention programs that target online hate should consider educating young people about problem-focused coping strategies, self-assertiveness, and media skills. Implications for future research are discussed.

## 1. Introduction

Hatred directed at members of groups due to their origin, race, gender, religion, or sexual orientation is by no means new. Comparatively new, however, is the practice of online hate, which has increased recently against the background of the proliferation of social media. Online hate can be expressed through offensive, insulting, or threatening texts or speech online, such as posts, comments, text messages, videos, and pictures. It directly or indirectly targets social groups based on gender, sexual orientation, disability, race, ethnicity, nationality, or religion, or individuals representing a certain group [1,2,3,4]. Online hate, as a specific form of online aggression, has similarities and differences to other forms of online aggression, such as online bullying. Both online hate and online bullying are used to intentionally harm and devalue a person or group using information and communication technology (ICT) [5]. Online bullying, however, is often described as a repeated activity that occurs over a longer period of time [6], whereas online hate may be a single act. Whereas online bullying can be directed at an individual, online hate is necessarily based on prejudicial views about social groups [5]. Given these conceptual similarities, some research has found an overlap between involvement in online hate and online bullying among adolescents [7]. However, this overlap is not complete; some adolescents engage in online hate but not in online bullying. Thus, to understand why some adolescents are involved in online hate, more research is needed. 

Online hate might be an especially important topic for adolescents because adolescents use ICT for the accomplishment of developmental tasks (i.e., identity formation and development of personal moral values and political opinions) [8]. Online hate might interfere with these processes; initial research on online hate and research in related fields, such as offline and online discrimination, showed that such experiences impact adolescents’ wellbeing and psychological functioning [1,9,10,11]. Since witnessing online hate is one of the most common ways to experience it [3,4], and past research has shown that even witnessing (online) aggression can negatively affect bystanders’ social relationships, resulting in toxic stress, and is correlated with perpetration [4,12,13,14], research on bystanders is important. Problem-focused coping strategies mitigate the detrimental effects of experiencing stressful situations on adolescents’ mental health [15,16]. The way in which bystanders manage stress and the negative emotions associated with online hate might influence whether adolescents are more or less likely to react aggressively, thereby becoming perpetrators themselves. However, no study has investigated whether problem-focused coping strategies moderate the association between being a bystander and perpetrator of online hate. In this study, we explored this relationship in detail. The results might help with developing intervention programs to reduce online hate among adolescents and mitigate its potential negative effects. The results might also help us to understand how adolescents can be supported to manage this emerging online risk.

### 1.1. Associations between Being a Bystander and Perpertrator of Online Hate

Adolescents can be directly involved in online hate, as victims or perpetrators, or indirectly involved as bystanders who observe online hate without being personally targeted by hateful material, comments, or posts [4,17]. Initial research on the prevalence rates of different forms of involvement in online hate suggests that the most common way to experience online hate is by witnessing it as a bystander. For example, in one study among 3500 adolescents and young adults from four countries, approximately 53% of American, 48% of Finnish, 39% of British, and 31% of German participants said they had witnessed cyberhate. In the same study, 16% of American, 10% of Finnish, 12% of British, and 4% of German participants reported that they have been personally targeted by cyberhate [18]. More recently, a study with French participants aged 11 to 20 years found that around 57% of participants had been exposed to online hate, approximately 10% were victimized through online hate on social networking sites, and 5% published or shared online hate material [19]. 

Several reasons support the suggestion that bystanders of online hate are more likely to be perpetrators of online hate. From a theoretical point of view, Bandura’s social learning theory posits that humans learn in part by imitating models, and that the status of models increases the likelihood that their behavior will be imitated [20]. Consequently, online hate perpetration might be explained by observational learning. Online hate is often conducted with the aim of influencing values within a peer group and establishing group identity [19]. Thus, when adolescents observe that their peers spread online hate and learn that online hate is appropriate or admirable behavior that can increase their social status and acceptance, bystanders might be more likely to participate and share, post, or forward hateful or denigrating online material against social groups. This assumption also seems reasonable because some research on (cyber)bullying has shown that being a bystander can increase moral disengagement and negative attitudes toward victims and reduce empathy for the victim [21,22,23]. Some empirical evidence shows that individuals tend to use more aggressive expressions in their online communication and interactions when their peers behave aggressively [12,14,21,24,25,26]. We therefore assumed that bystanders to online hate have higher odds of being perpetrators of online hate. 

Until now, only one study investigated which factors influence the relationship between being a bystander to and perpetrator of online hate among adolescents. In a study with around 1500 German adolescents, higher levels of toxic online disinhibition increased the likelihood of bystanders of online hate being perpetrators of online hate [4]. Another explanatory mechanism in this relationship is the lack of appropriate coping strategies. Thus far, the role of certain coping strategies in this relationship is largely unknown.

### 1.2. Adolescents’ Online Hate Coping Stratgies 

When people experience a stressful interaction, they attempt to mitigate or eliminate the negative effects of this event. This process is referred to as coping, which is defined as the effort to manage stress and the subsequent emotions caused by such stress [27]. Being able to cope efficiently with interpersonal conflicts has been recognized as an important component of social skills, which in turn can be considered as a protective factor against aggressive behavior [28]. 

Little empirical research has been conducted on how adolescents respond to online hate. One study revealed that adolescents use different actions in relation to online hate. The most frequent reaction if they see something hateful on the internet is to ignore it (43%); followed by reporting it to the social networking website, app, game, or website (20%); speaking to a friend about it (16%); blocking the online hate perpetrator (15%); telling a parent or another adult (11%); replying publicly (9%) or privately (3%) to the online hate perpetrator; telling a teacher or other professional (2%); and reporting the behavior to the police (2%) [1]. How adolescents respond to online hate is also influenced by whether they were directly targeted. If adolescents feel directly targeted they are more likely to take action, compared to online hate that is not directly targeting the person [1]. 

In cyberbullying research, coping strategies have been investigated thoroughly. This research mainly focused on victims of cyberbullying. Based on a systematic literature review, Perren et al. [29] concluded that four main groups of strategies can be distinguished: reactions toward the perpetrator (e.g., retaliation, confronting the perpetrator), technical coping (e.g., block the perpetrator, report online hate material), supportive strategies (e.g., talk to peers, parents, adults), and avoidant and emotion-focused strategies (e.g., ignoring). Machackova et al. [30] concluded that, by summarizing research on adolescents’ use of coping strategies against cyberbullying and their perceived effectivity, assertive strategies are rarely employed and are often evaluated as not useful, whereas technical coping strategies are frequently used and evaluated as effective. 

Both assertive and technical coping strategies can be considered examples of problem-focused coping strategies. When exposure to online hate is seen as a stressor, the question arises as to whether problem-focused coping strategies can mitigate negative outcomes (i.e., the externalization of problem behaviors, such as perpetrating online hate). Problem-focused coping strategies help people to adjust better to threatening situations [31]. More specifically, research has shown that problem-focused coping strategies mitigate the negative consequences of (cyber)victimization on psychological functioning and wellbeing [15,16,32,33]. We therefore expected that problem-focused coping (i.e., assertive and technical coping) would reduce the likelihood of bystanders becoming perpetrators. 

### 1.3. Present Study

We aimed to contribute to the existing knowledge about the relationship between witnessing and perpetrating online hate among adolescents. To enhance our understanding of this relationship, assertive and technical coping were investigated as moderators. The results might help to inform effective intervention and prevention initiatives in the field of media education. In contrast to previous research on exposure and perpetration of online aggression, this is the first study to examine this relationship for online hate among adolescents from eight countries. To guide this purpose, we hypothesized that:

**Hypothesis 1 (H1).**  *Being a bystander to online hate is positively related with being a perpetrator of online hate.*

**Hypothesis 2 (H2).**  *Higher levels of assertive coping weaken the association between being a bystander to and a perpetrator of online hate.*

**Hypothesis 1 (H3).**  *Higher levels of technical coping weaken the association between being a bystander to and a perpetrator of online hate.*

## 2. Materials and Methods 

### 2.1. Participants

A total of 6829 adolescents (age = 12–18 years; Mean age (M*_age_*) = 14.93; SD = 1.64; female: *n* = 3442, 50.8%) participated in this study. The study sample included participants from Cyprus (*n* = 221; age= 12–18 years; M*_age_* = 14.49; SD = 1.48; female: 68%), Germany (*n* = 1480; age = 12–17 years; M*_age_* = 14.21; SD = 1.23; female: 50.3%), Greece (*n* = 670; age = 15–18 years; M*_age_* = 16.49; SD = 1.12; female: 53.6%), India (*n* = 1121; age = 13–18 years; M*_age_* = 15.37; SD = 1.48; girls: 45%), South Korea (*n* = 756; age = 12–17 years; M*_age_* = 14.73; SD = 1.23; female: 49.8%), Spain (*n* = 1018; age range = 12–18 years; M*_age_* = 14.29; SD = 1.64; female: 51.7%), Thailand (*n* = 716; age = 13–18 years; M*_age_* = 15.68; SD = 1.70; female: 52.8%), and the United States (*n* = 847; age = 12–18 years; M*_age_* = 14.79; SD = 1.80; female: 50.7%). 

### 2.2. Measures

#### 2.2.1. Online Hate Involvement

Participants were provided the following definition of online hate to increase validity of responses: 

Online hate describes the usage of information and communication technologies (e.g., WhatsApp, Facebook, Instagram, Twitter) to offend and hurt somebody because of their race, gender, ethnic group, nationality, disability, sexual orientation, or religion. It can be either targeted directly at a person or group, or generally shared online. Online hate can be offensive, mean or threatening and can be expressed through degrading writings or speech online such as posts, comments, text messages, videos or pictures.

Two items were used to measure being a bystander to and perpetrator of online hate. These items were adopted from past research conducted by Hawdon et al. [3]. For measuring being a bystander to online hate, participants were asked: “How often in the past 12 months have you observed hateful or degrading writing or speech online, which inappropriately attacks certain groups of people or individuals because of their sex, religious affiliation, race, or sexual orientation?” For measuring online hate perpetration, participants were asked: “How often in the past 12 months have you posted hateful or degrading writing or speech online, which inappropriately attacks certain groups of people or individuals based on their sex, religious affiliation, race, or sexual orientation?” All items had to be answered on five-point scales ranging from 0 (never) to 4 (very frequently). 

#### 2.2.2. Assertive and Technical Coping

Assertive and technical coping with online hate was assessed using two scales of the Coping with Cyberbullying Questionnaire developed by Sticca et al. [34]. In our adaptation of this instrument, participants were presented a description of the following scenario to the students: “A person has expressed hateful or degrading writing or speech online through posts, comments, text messages, videos, or pictures, which inappropriately attacked you because of your race, gender, ethnic group, sexual orientation, or religion via chats or social networks (e.g., Facebook, Instagram, WhatsApp)”. After the description, we asked participants to imagine how they would cope with cyberhate victimization. For assertive coping, they rated how likely they would be to use the following four strategies: “I would let the person know that I do not find it funny at all”, “…let the person know that their behavior is not acceptable at all”, “...tell the person to stop it”, and “…ask the person why they are doing this”. For technical coping, participants were asked to rate how likely they would be to use the following actions: “I would pay more attention to who has access to my data”, “...block that person so that they cannot contact me anymore”, and “…save messages/pictures as evidence (e.g., copies or screenshots)”. Participants rated how likely they were to use each action on a scale ranging from “definitely not” (0) to “definitely” (3). The Cronbach’s α values for assertive coping were: 0.91 for Cyprus, 0.90 for Germany, 0.88 for Greece, 0.90 for India, 0.81 for South Korea, 0.86 for Spain, 0.95 for Thailand, 0.75 for the US, and 0.88 for the total sample. The Cronbach’s α values for technical coping were: 0.81 for Cyprus, 0.83 for Germany, 0.78 for Greece, 0.78 for India, 0.69 for South Korea, 0.74 for Spain, 0.82 for Thailand, 0.72 for the U.S., and 0.83 for the total sample. 

As control variables, participants were asked for their age and sex to determine demographic characteristics.

### 2.3. Procedures

In all countries, approval was obtained by the researchers’ Institutional Review Board from their respective university and/or educational authorities, except for India. In India, it was only required for school principals and parents to provide their consent for adolescents’ participation. The Helsinki ethics protocol was followed throughout the conduct of this study [35]. To recruit schools, research personnel contacted school principals through emails or phone calls to discuss the aims of the study and how adolescents could participate. After classroom announcements, parental permission slips were sent home with adolescents for them to provide to their parent(s) or guardian(s). Parental permission slips were then returned to adolescents’ classrooms. Data were collected at adolescents’ schools during regular school hours or extracurricular activities. During the data collection session, adolescents provided their own consent to participate in the study. The translation procedure from English into the target language of the research instrument was uniformly regulated in Cyprus, Greece, Korea, Spain, and Thailand and was completed as recommended: translation of the original instrument into the target language and then back-translation by another person who had not seen the original questionnaire. The new translation was then compared with the original instrument [36]. The existing English version of the research instrument was used in India and the U.S., and the existing German version in Germany.

### 2.4. Data Analyses

Descriptive statistics were used to determine means, standard deviations, and frequency rates of the study’s variables. Pearson’s *r* correlations were used to investigate the bivariate associations among variables. Binary logistic regressions were used to investigate the influence of demographic variables on being a bystander to online hate and being a perpetrator of online hate. A multiple regression analysis was used to examine the regression-based double moderated model. To perform this analysis, we used the PROCESS macro for SPSS 24 (SPSS Inc., Chicago, IL, USA) developed by Hayes [37], with 5000 bias-corrected bootstrap samples. Being a bystander to online hate was the independent variable, assertive and technical coping were the moderators, and online hate perpetration was the dependent variable. Participants’ age, sex, and country of origin were included as control variables. Cohen’s *f^2^* was used as an effect size for multiple regression. According to Cohen [38], *f^2^* ≥ 0.10, *f^2^* ≥ 0.25, and *f^2^* ≥ 0.40 represent small, medium, and large effect sizes, respectively. Multicollinearity diagnostics between the study’s main variables were assessed and were within an acceptable range (Table 1).

## 3. Results

### 3.1. Descriptive Statistics 

Overall, 49.1% (*n* = 3308) of participants stated that they witnessed at least one incident of hateful or degrading writing or speech online. Concerning the frequencies, 50.9% (*n* = 3436) reported they had never witnessed online hate, 15.7% (*n* = 1060) reported witnessing online hate very rarely, 15.7% (*n* = 1060) occasionally, 9.8% (*n* = 661) frequently, and 7.8% (*n* = 527) very frequently. Regarding online hate perpetration, 14.2% (*n* = 961) of participants reported that they had posted hateful or degrading writing or speech online at least once. A total of 85.8% (*n* = 5785) reported they had never posted online hate, 9.1% (*n* = 614) reported posting online hate very rarely, 3.5% (*n* = 235) occasionally, 1.0% (*n* = 69) frequently, and 0.6% (*n* = 43) very frequently. 

Means, standard deviations, and percentages for online hate, and means and standard deviations for assertive and technical coping are presented in Table 2. Frequency rates of being bystanders to online hate varied between 31% among Indian adolescents and 68.5% among Spanish adolescents, and between 4.2% among Korean adolescents and 32.2% among Thai adolescents, for being perpetrators of online hate. Means of assertive coping varied between *M* = 1.39 among Thai adolescents and *M* = 2.21 among Spanish adolescents, and for technical coping between *M* = 0.70 among American adolescents and *M* = 2.45 for Cypriot adolescents. 

### 3.2. Differences in Online Hate by Country, Age, and Sex

To investigate differences by country of origin, age, and sex, two binary logistic regressions were conducted, with either being a bystander to or being a perpetrator of online hate as the outcome variable, and country of origin, age, and sex as predictors. The association between demographic variables and being a bystander to online hate is shown in Table 3. Regarding country of origin, being Cypriot (odds ratio (OR) = 0.424, 95% CI = 0.315–0.572, *p* < 0.001), Greek (OR = 0.693, 95% CI = 0.566–0.849, *p* < 0.001), Indian (OR = 0.326, 95% CI = 0.275–0.387, *p* < 0.001), Korean (OR = 0.511, 95% CI = 0.426–0.613, *p* < 0.001), and American (OR = 0.448, 95% CI = 0.373–0.539, *p* < 0.001) were associated with lower odds of being a bystander to online hate compared with being German. By contrast, Spanish (OR = 1.85, 95% CI = 1.56–2.20, *p* < 0.001) and Thai adolescents (OR = 1.24, 95% CI = 1.02–1.51, *p* = 0.027) showed higher odds of witnessing online hate compared to German adolescents. Age decreased the odds of witnessing online hate (OR = 1.17, 95% CI = 1.13–1.21, *p* < 0.001). Being male (OR = 0.701, 95% CI = 0.634–0.776, *p* < 0.001) was associated with lower odds of witnessing online hate. 

Table 4 shows the results of the binary logistic regression for online hate perpetration. Regarding country of origin, Cypriot (OR = 0.404, 95% CI = 0.209–0.780, *p* = 0.007), Korean (OR = 0.333, 95% CI = 0.225–0.493, *p* < 0.001), and Spanish (OR = 0.664, 95% CI = 0.500–0.882, *p* = 0.005) adolescents showed lower odds of being perpetrators of online hate compared to German adolescents. However, being Thai (OR = 1.23, 95% CI = 2.70–4.37, *p* < 0.001) or American (OR = 2.57, 95% CI = 2.03–3.25, *p* < 0.001) was associated with higher odds of being a perpetrator of online hate compared to being German. Increasing age (OR = 1.07, 95% CI = 1.02–1.12, *p* = 0.002) and being male (OR = 1.59, 95% CI = 1.37–1.84, *p* < 0.001) were associated with higher odds of committing online hate. 

### 3.3. Moderation Analysis 

The regression model was significant and accounted for 15% of the variance in online hate perpetration (*F* (14, 6469) = 78.23, *p* < 0.001, *R^2^* = 0.15), indicating a small effect (Cohen’s *f^2^* = 0.17). A number of hypothesized predictors significantly explained variance in online hate perpetration, which were significant correlates of online hate perpetration (Table 5). This included being a bystander to online hate (*β* = 0.307, *p* < 0.001), assertive coping (*β* = −0.063, *p* < 0.001), and technical coping (*β* = −0.052, *p* < 0.001; Table 5). Country of origin, age, and sex were included as control variables.

**Figure 1:**
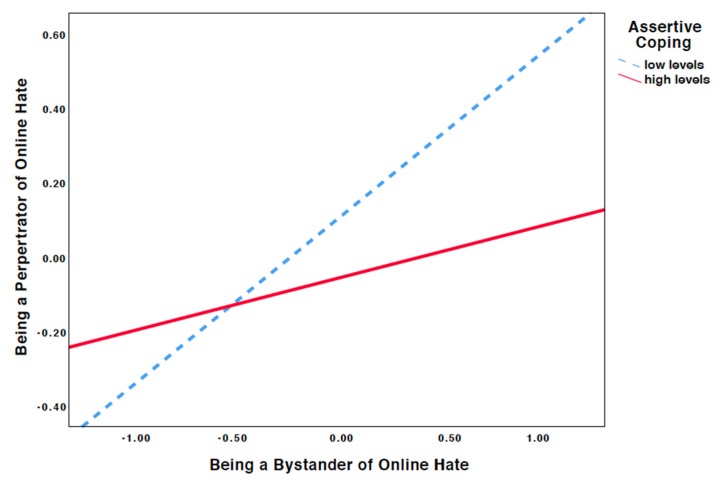
Simple slopes equations of the regression of online hate bystanders on online hate perpetrators at high and low levels of assertive coping.

Significant moderation effects were found between being a bystander to online hate and assertive coping when predicting online hate perpetration (*β* = −0.076, standard error (SE) = 0.017, *p* < 0.001). Probing the interaction further revealed that bystanders to online hate reported more online hate perpetration when they reported lower levels of assertive coping (*β* = 0.439, SE = 0.018, *p* < 0.001 at −1 SD) and less frequent online hate perpetration when they reported higher levels of toxic online disinhibition (β = 0.138, SE = 0.016, *p* < 0.001 at +1 SD*;* Figure 1). 

**Figure 2:**
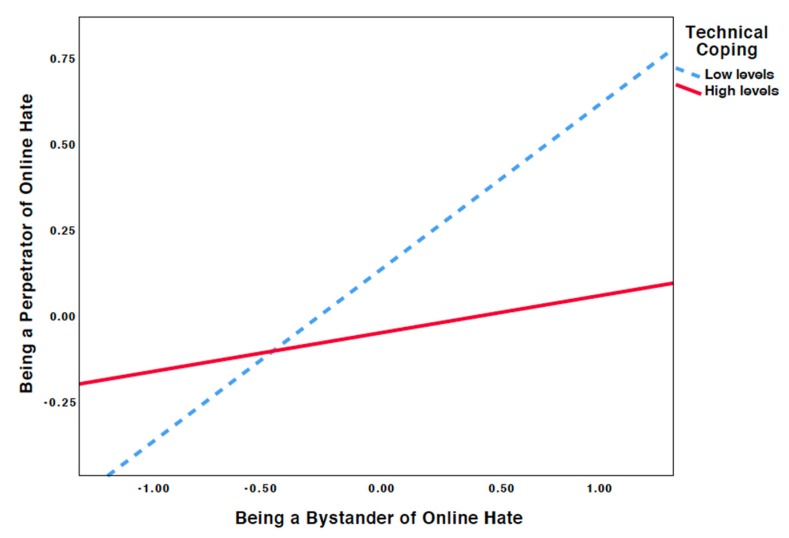
Simple slope equations of the regression of online hate bystanders on online hate perpetrators at high and low levels of technical coping.

Consistent patterns were found for the moderation of being a bystander to online hate by technical coping when predicting online hate perpetration (*β* = −0.151, SE = 0.015, *p* < 0.001). Probing the interaction further revealed that bystanders to online hate reported more online hate perpetration when they reported lower levels of assertive coping (*β* = 0.489, SE = 0.019, *p* < 0.001 at −1 SD) and less frequent online hate perpetration when they reported higher levels of toxic online disinhibition (*β* = 0.110, SE = 0.016, *p* < 0.001 at +1 SD*;* Figure 2). 

## 4. Discussion

The online world has become an important place for many adolescents around the world. ICT offers many opportunities and tools for adolescents to perform developmental tasks [8]. With the worldwide increase in populism and extremism, however, new online risks, such as online hate, have emerged [4,19,39]. To inform the development of online hate prevention programs, we examined the relationship between being a bystander to and a perpetrator of online hate, and whether assertive and technical coping moderates this relationship, in a large sample of adolescents from eight countries. 

Overall, 49% had witnessed online hate at least once and 14% reported having perpetrated online hate within the last 12 months at least once. The results support findings from past research: Being a bystander to online hate is much more likely than playing an active role [3]. Concerning differences in country of origin, our findings are difficult to compare with past research because not much is known about national differences related to online hate among adolescents. In our study, the frequency rates varied between 31% in India and 68.5% in Spain for witnessing online hate, and between 4.2% in Korea and 32.2% in Thailand for perpetrating online hate. These findings show that adolescents’ exposure to online hate is common in different regions and different cultures around the world. Compared to German adolescents, Cypriot, Greek, Indian, Korean, and American adolescents were less likely to be exposed to online hate, and Spanish and Thai adolescents were more likely. Cypriot, Korean, and Spanish adolescents showed lower odds of being perpetrators of online hate, and Thai and American adolescents had higher odds compared to German adolescents. Understanding national differences in the frequency rates of online hate is complex and most likely influenced by individual and wider societal factors (e.g., the presence of antihate speech laws, terrorist attacks) [18,39,40]. Age was positively associated with being a bystander to and perpetrator of online hate, indicating that adolescents become, with increasing age, more likely to witness online hate and also post or forward hateful or denigrating material online. Regarding sex, boys showed lower odds of seeing online hate and higher odds of perpetrating online hate compared to girls. 

We found support for our hypothesis that bystanders to online hate are more likely to be perpetrators of online hate (H1). This relationship might be explained through observational learning, dynamic group processes, and lack of appropriate coping strategies. Through witnessing online hate, some bystanders might think that this is appropriate or even admirable behavior, which could increase their social status and acceptance. Related research found that exposure to (cyber)bullying can lead to high moral disengagement, lower levels of empathic responsiveness, and negative attitudes toward victims [21,22,23], which in turn can increase the likelihood of perpetration. Further research is needed to understand whether the relationship between being a bystander to and perpetrator of online hate could also be explained by desensitization effects, in terms of lower empathic responsiveness and more positive attitudes toward online hate. Studying this relationship is important as it might advance the understanding of online hate among adolescents.

Our finding that witnessing and perpetration of online hate are positively correlated is in line with past research, which found that individuals were more likely to commit aggression when their peers behave aggressively, and also in line with longitudinal studies that have shown that being a bystander predicts being a perpetrator in (cyber)bullying [12,14,21,24,25,26]. With this study, we have extended the literature on the relationship between being a bystander to and perpetrator of online hate.

As witnessing online hate is also positively associated with online hate perpetration, empowering adolescents with the knowledge, skills, and confidence to manage online hate is essential. Currently, adolescents often do not know the differences between free speech and hate speech, when online hate breaks the law, and how it can be reported (or they lack confidence to do so) [1]. When bystanders remain passive, however, this might provide the impression that they indirectly and silently support online hate, which could normalize online hate and encourage adolescents who perpetrate online hate in their aggressive behavior, while demotivating adolescents who observe online hate to engage in counter speech.

Consistent with our second hypothesis, we found that higher levels of assertive coping weakened the association between being a bystander to and being a perpetrator of online hate (H2). This finding indicates that assertively responding to exposure to online hate might reduce the likelihood of an aggressive response. We found that higher levels of technical coping weakened the association between being a bystander to and being a perpetrator of online hate, providing support for our third hypothesis. Taken together, the findings are in line with previous research on (cyber)bullying, which showed the mitigating effects of problem-focused coping strategies on the association between (cyber)bullying and psychological problems [15,16,32,33]. Comparing the magnitude of the moderation effect of assertive coping with being a bystander to online hate (β = −0.076) and technical coping with being a bystander to online hate (β = −0.151) to predict online hate perpetration, our findings are somewhat in line with the conclusion of Machackova et al. [30], who described technical coping as more effective than assertive coping in managing cyberbullying. We also found that both assertive and technical coping are negatively related with being a perpetrator of online hate. This finding is in line with other research that found that the inability to cope efficiently with conflict could be considered a lack of social skills and a risk factor for perpetrating aggression [28]. Future research should investigate whether perpetrators show higher endorsement of less constructive coping strategies (e.g., revenge) over more constructive coping strategies. Together, our findings highlight the need for assertiveness training and media skills training in online hate prevention programs.

Assertiveness training programs could aim to empower adolescents to resist group pressure, not join in online hate, and defend oneself without being offensive to others. This kind of training could also increase adolescents’ self-efficacy when intervening in online hate. Media skills training should teach adolescents to pay more attention to who is allowed access to their data, how to block people who are sharing online hate material, how to save messages/pictures as evidence (e.g., copies or screenshots), and how to report online hate material to social networking websites. Ethical media literacy might also be beneficial for reducing online hate. Adolescents with strong ethical media competence are better able to assess their behavior and the resulting consequences [41]. Adolescents with strong ethical media literacy are less vulnerable to online victimization due to their reflected use of digital media (e.g., with regard to online disclosure of private information) [41]. 

To increase the positive effects of these programs, another option is the introduction of peer-mentoring training groups, in which peers are used as trainers and role models. Some evidence shows that peer-tutoring intervention programs can be an effective approach to reducing cyberbullying among adolescents [42]. However, before intervention and prevention programs on online hate can be developed, more knowledge about its correlates is needed to understand why adolescents engage in online hate.

Despite the promising results, the current study also has limitations. The most important limitation is the cross-sectional nature of the data. Future research should investigate these variables at several points in time. This improvement will enable the determination of the temporal ordering of the variables and the moderation effects examined in this study. Single-item measures were used for being a bystander to and perpetrator of online hate. Follow-up research should use validated scales to overcome reliability and validity assessment issues. Despite the large sample size used, the sample cannot be considered representative of adolescents from the participating eight countries. Consequently, researchers should conduct studies that include representative samples from these countries to increase the generalizability of this research. Studies should also be conducted based on diverse samples, including ones that vary by, for example, educational level, sexual identity, religious affiliation or racial/ethnic group. These samples might also allow the investigation of different forms of online hate in more detail (i.e., homophobic, xenophobic, and Islamophobic). Another limitation of the present study is the exclusive reliance on self-reports. Thus, some of the observed associations might be biased through shared method variance. We only included two problem-focused coping strategies: assertive and technical coping. Follow-up research should include a wider range of coping strategies to understand whether maladaptive coping strategies increase the association between being a bystander to and perpetrator of online hate. The coping strategies were measured for a hypothetical online hate scenario. Thus, the reported coping strategies might be based on behavioral intentions rather than on real behavior. Future research should use a recalled, real online hate situation to investigate how adolescents actually cope with online hate. Finally, if adolescents lack the awareness of the classification of their behavior as online hate, they might also underreport online hate.

## 5. Conclusions

Our study is among the first to elucidate the moderating effects of two problem-focused coping strategies on the relationship between being a bystander to and being a perpetrator of online hate. Our findings further advance the understanding of the involvement of adolescents in online hate behavior in eight countries. We found that assertive and technical coping strategies are negatively related with being a perpetrator of online hate. The results highlight the importance of problem-focused coping strategies in the relationship between being a bystander to and perpetrator of online hate. Future studies should focus on developing a better understanding of how different coping strategies (i.e., emotion-focused vs. maladaptive coping strategies) impact this relationship. The current findings indicate a need for media pedagogues to educate adolescents to manage online hate by using assertive and technical strategies. More attention should be paid to developing intervention programs that focus on coping strategies to help to mitigate the likelihood that adolescents become involved in online hate.

## Figures and Tables

**Table 1:** Bivariate correlations between online hate bystanders, online hate perpetrators, assertive coping, and technical coping.

Variable	1	2	3	4
1. Online hate bystanders	-	-	-	-
2. Online hate perpetrators	0.27 **	-	-	-
3. Assertive Coping	0.16 **	−0.08 **	-	-
4. Technical Coping	0.19 **	−0.11 **	0.58 **	-

** *p* < 0.001.

**Table 2:** Descriptive statistics for online hate bystanders, online hate perpetrators, assertive coping, and technical coping.

Country	Bystanders to Online Hate			Perpetrators of Online Hate			Assertive Coping		Technical Coping	
	Mean	SD	*%*	Mean	SD	*%*	Mean	SD	Mean	SD
Cyprus	0.75	1.18	35.7	0.05	0.28	4.6	1.96	1.02	2.45	0.81
Germany	1.15	1.32	53.7	0.19	0.62	11.3	1.67	1.03	1.90	1.04
Greece	1.35	1.46	54	0.19	0.63	11.1	1.94	0.95	2.41	0.77
India	0.64	1.13	31.4	0.24	0.63	15.3	1.50	1.19	1.53	1.09
South Korea	0.89	1.26	39.3	0.07	0.36	4.2	1.86	0.83	2.14	0.77
Spain	1.65	1.44	68.5	0.12	0.48	7.8	2.21	0.83	2.41	0.76
Thailand	1.37	1.26	65.0	0.47	0.77	32.2	1.39	1.17	1.25	1.06
USA	0.63	1.01	36.2	0.34	0.70	24.5	1.71	0.86	0.70	0.78
Total	1.08	1.32	49.1	0.22	0.61	14.2	1.76	1.03	1.80	1.08

**Table 3:** Demographic predictors of being a bystander to online hate. OR, odds ratio.

Factor	B	*p*-Value	OR	95% CI for OR	
				Lower	Upper
Constant	−1.605	<0.001			
Being Cypriot ^1^	−0.857	<0.001	0.424	0.315	0.572
Being Greek ^1^	−0.366	<0.001	0.693	0.566	0.849
Being Indian ^1^	−1.211	<0.001	0.326	0.275	0.387
Being Korean ^1^	−0.672	<.0.001	0.511	0.426	0.613
Being Spanish ^1^	0.619	<0.001	1.858	1.566	2.204
Being Thai ^1^	0.220	0.027	1.246	1.026	1.515
Being American ^1^	−0.803	<0.001	0.448	0.373	0.539
Age	0.161	<0.001	1.175	1.134	1.216
Being male ^2^	−0.355	<0.001	0.701	0.634	0.776

Note: ^1^ Reference category: being German; ^2^ reference category: being female. The online hate variable was dichotomized (never = no; very rarely–very frequently = yes).

**Table 4:** Demographic predictors of being a perpetrator of online hate.

Factor	B	*p*-Value	OR	95% CI for OR	
				Lower	Upper
Constant	−3.807	<0.001			
Being Cypriot ^1^	−0.906	0.007	0.404	0.209	0.780
Being Greek ^1^	−0.184	0.250	0.832	0.608	1.138
Being Indian ^1^	0.240	<0.050	1.271	1.000	1.614
Being South Korean ^1^	−1.100	<0.001	0.333	0.225	0.493
Being Spanish ^1^	−0.409	0.005	0.664	0.500	0.882
Being Thai ^1^	1.235	<0.001	3.439	2.706	4.370
Being American ^1^	0.946	<0.001	2.575	2.037	3.255
Age	0.072	0.002	1.075	1.026	1.126
Being male ^2^	0.465	<0.001	1.592	1.378	1.840

Note: ^1^ Reference category: being German; ^2^ reference category: being female. The online hate variable was dichotomized (never = no; very rarely–very frequently = yes).

**Table 5:** Coefficients of the model predicting online hate perpetration.

Model	*β* (*)	SE	*t*	*p*
Constant	−0.412 (−0.646 to 0.178)	0.119	−3.46	<0.001
Online hate bystanders	0.307 (0.282 to 0.331)	0.012	24.64	<0.001
Assertive Coping	−0.063 (−0.093 to −0.034)	0.014	−4.29	<0.001
Technical Coping	−0.052 (−0.085 to −0.018)	0.017	−3.06	0.002
OHB × Assertive Coping	−0.076 (−0.105 to −0.047)	0.014	−5.18	<0.001
OHB × Technical Coping	−0.151 (−0.180 to −0.120)	0.015	−9.77	<0.001

Note: OHB = online hate bystanders; * 95% BCa = bootstrap confidence intervals based on 5000 samples; SE = standard error.
