# Correction: Wachs, S., et al. Associations between Witnessing and Perpetrating Online Hate in Eight Countries: The Buffering Effects of Problem-Focused Coping. *Int. J. Environ. Res. Public Health* 2019, *16*, 3992

**DOI:** 10.3390/ijerph18052609

**Published:** 2021-03-05

**Authors:** Sebastian Wachs, Michelle F. Wright, Ruthaychonnee Sittichai, Ritu Singh, Ramakrishna Biswal, Eun-mee Kim, Soeun Yang, Manuel Gámez-Guadix, Carmen Almendros, Katerina Flora, Vassiliki Daskalou, Evdoxia Maziridou

**Affiliations:** 1 Department of Educational Studies, University of Potsdam, 14476 Potsdam, Germany; 2 Department of Psychology, Pennsylvania State University, University Park, PA 16802, USA; mfw5215@psu.edu; 3 Faculty of Social Studies, Masaryk University, 60200 Brno, Czech Republic; 4 Kids and Youth Development Research Unit, Research Center for Educational Innovations and Teaching and Learning Excellence, Faculty of Humanities and Social Sciences, Prince of Songkla University, Muang, Pattani 94000, Thailand; ruthaychonnee.s@psu.ac.th; 5 Department of Human Development and Family Studies, G.B. Pant University of Agriculture and Technology, Pantnagar, Uttarakhand 263145, India; ritu.singh07@gmail.com; 6 Department of Humanities and Social Sciences, National Institute of Technology, Rourkela 769008, India; rkbpsych@gmail.com; 7 Department of Communication, Seoul National University, Seoul 08826, Korea; eunmee@snu.ac.kr (E.-m.K.); soeun022@snu.ac.kr (S.Y.); 8 Department of Biological and Health Psychology, Autonomous University of Madrid, 28049 Madrid, Spain; manuel.gamez@uam.es (M.G.-G.); carmen.almendros@uam.es (C.A.); 9 Department of Psychology, Neapolis University Pafos, Pafos 8042, Cyprus; K.flora@nup.ac.cy; 10 Department of Psychology, Aristotle University of Thessaloniki, 54124 Thessaloniki, Greece; vassilikida@yahoo.gr (V.D.); evamazirid@gmail.com (E.M.)

The authors wish to add the following corrections to their paper published in the International Journal of Environmental Research and Public Health [1].

In the second paragraph of Section 3.3, “toxic online disinhibition” should be replaced with “assertive coping”. Thus, the sentence should read with “Probing the interaction further revealed that bystanders to online hate reported more online hate perpetration when they reported lower levels of assertive coping (β = 0.439, SE = 0.018, *p* < 0.001 at −1 SD) and less frequent online hate perpetration when they reported higher levels of assertive coping (β = 0.138, SE = 0.016, *p* < 0.001 at +1 SD; Figure 1).”

In the third paragraph of Section 3.3, “assertive coping” and “toxic online disinhibition” should both be replaced with “technical coping”. Thus, the sentence should read “Probing the interaction further revealed that bystanders to online hate reported more online hate perpetration when they reported lower levels of technical coping (β = 0.489, SE = 0.019, p < 0.001 at −1 SD) and less frequent online hate perpetration when they reported higher levels of technical coping (β = 0.110, SE = 0.016, p < 0.001 at +1 SD; Figure 2).”

We apologize for any inconvenience caused to the readers by this change. The changes do not affect the scientific results. The published version will be updated on the article webpage, with a reference to this Erratum.
